# Supplementary material for: Unveiling the Bioactive Potential of the Invasive Jellyfish Phyllorhiza punctata Through Integrative Transcriptomic and Proteomic Analyses
Source: Biomolecules. 2025 Aug 4;15(8):1121. doi: 10.3390/biom15081121 (PMC12383608; doi:10.3390/biom15081121)
Supplement: Supplementary file 1 [file biomolecules-15-01121-s001.zip › Suplementary Material/FileS3/PEPFOLD4/XP_065062463_9914/clusters.html]

 PEP-FOLD clusters


Download archive of all models  here   

|  |  |  |
| --- | --- | --- |
| # | file | sOPEP |
| # cluster 1 - Size: 6 |  |  |
|  | XP\_065062463\_9914-model1 | -30.0769 |
|  | XP\_065062463\_9914-model1.1 | -29.9617 |
|  | XP\_065062463\_9914-model1.2 | -29.9238 |
|  | XP\_065062463\_9914-model1.3 | -29.8917 |
|  | XP\_065062463\_9914-model1.4 | -29.6336 |
|  | XP\_065062463\_9914-model1.5 | -29.2055 |
| # cluster 2 - Size: 2 |  |  |
|  | XP\_065062463\_9914-model2 | -25.8278 |
|  | XP\_065062463\_9914-model2.1 | -24.9696 |
| # cluster 3 - Size: 3 |  |  |
|  | XP\_065062463\_9914-model3 | -22.555 |
|  | XP\_065062463\_9914-model3.1 | -22.4505 |
|  | XP\_065062463\_9914-model3.2 | -22.3569 |
| # cluster 4 - Size: 17 |  |  |
|  | XP\_065062463\_9914-model4 | -21.0995 |
|  | XP\_065062463\_9914-model4.1 | -20.8214 |
|  | XP\_065062463\_9914-model4.2 | -20.6193 |
|  | XP\_065062463\_9914-model4.3 | -20.6124 |
|  | XP\_065062463\_9914-model4.4 | -20.5326 |
|  | XP\_065062463\_9914-model4.5 | -20.461 |
|  | XP\_065062463\_9914-model4.6 | -20.4512 |
|  | XP\_065062463\_9914-model4.7 | -20.1353 |
|  | XP\_065062463\_9914-model4.8 | -20.0024 |
|  | XP\_065062463\_9914-model4.9 | -19.8036 |
|  | XP\_065062463\_9914-model4.10 | -19.6016 |
|  | XP\_065062463\_9914-model4.11 | -19.5925 |
|  | XP\_065062463\_9914-model4.12 | -19.2061 |
|  | XP\_065062463\_9914-model4.13 | -19.087 |
|  | XP\_065062463\_9914-model4.14 | -18.5915 |
|  | XP\_065062463\_9914-model4.15 | -18.2574 |
|  | XP\_065062463\_9914-model4.16 | -18.1506 |
| # cluster 5 - Size: 10 |  |  |
|  | XP\_065062463\_9914-model5 | -20.4721 |
|  | XP\_065062463\_9914-model5.1 | -20.3479 |
|  | XP\_065062463\_9914-model5.2 | -20.337 |
|  | XP\_065062463\_9914-model5.3 | -20.2108 |
|  | XP\_065062463\_9914-model5.4 | -20.0569 |
|  | XP\_065062463\_9914-model5.5 | -19.9125 |
|  | XP\_065062463\_9914-model5.6 | -19.9111 |
|  | XP\_065062463\_9914-model5.7 | -19.9029 |
|  | XP\_065062463\_9914-model5.8 | -19.8885 |
|  | XP\_065062463\_9914-model5.9 | -17.7882 |
| # cluster 6 - Size: 11 |  |  |
|  | XP\_065062463\_9914-model6 | -19.7295 |
|  | XP\_065062463\_9914-model6.1 | -19.6236 |
|  | XP\_065062463\_9914-model6.2 | -19.5526 |
|  | XP\_065062463\_9914-model6.3 | -18.7631 |
|  | XP\_065062463\_9914-model6.4 | -18.7524 |
|  | XP\_065062463\_9914-model6.5 | -18.1617 |
|  | XP\_065062463\_9914-model6.6 | -18.0093 |
|  | XP\_065062463\_9914-model6.7 | -17.6528 |
|  | XP\_065062463\_9914-model6.8 | -17.2685 |
|  | XP\_065062463\_9914-model6.9 | -17.2422 |
|  | XP\_065062463\_9914-model6.10 | -17.2308 |
| # cluster 7 - Size: 3 |  |  |
|  | XP\_065062463\_9914-model7 | -18.5504 |
|  | XP\_065062463\_9914-model7.1 | -18.0913 |
|  | XP\_065062463\_9914-model7.2 | -17.9843 |
| # cluster 8 - Size: 2 |  |  |
|  | XP\_065062463\_9914-model8 | -18.0992 |
|  | XP\_065062463\_9914-model8.1 | -17.4268 |
| # cluster 9 - Size: 1 |  |  |
|  | XP\_065062463\_9914-model9 | -18.0397 |
| # cluster 10 - Size: 2 |  |  |
|  | XP\_065062463\_9914-model10 | -17.9453 |
|  | XP\_065062463\_9914-model10.1 | -17.9303 |
| # cluster 11 - Size: 18 |  |  |
|  | XP\_065062463\_9914-model11 | -17.9203 |
|  | XP\_065062463\_9914-model11.1 | -17.7981 |
|  | XP\_065062463\_9914-model11.2 | -17.5788 |
|  | XP\_065062463\_9914-model11.3 | -17.5755 |
|  | XP\_065062463\_9914-model11.4 | -17.426 |
|  | XP\_065062463\_9914-model11.5 | -17.3781 |
|  | XP\_065062463\_9914-model11.6 | -17.2631 |
|  | XP\_065062463\_9914-model11.7 | -17.1425 |
|  | XP\_065062463\_9914-model11.8 | -17.0477 |
|  | XP\_065062463\_9914-model11.9 | -16.9841 |
|  | XP\_065062463\_9914-model11.10 | -16.9348 |
|  | XP\_065062463\_9914-model11.11 | -16.9021 |
|  | XP\_065062463\_9914-model11.12 | -16.8873 |
|  | XP\_065062463\_9914-model11.13 | -16.8769 |
|  | XP\_065062463\_9914-model11.14 | -16.8316 |
|  | XP\_065062463\_9914-model11.15 | -16.717 |
|  | XP\_065062463\_9914-model11.16 | -16.7161 |
|  | XP\_065062463\_9914-model11.17 | -16.3385 |
| # cluster 12 - Size: 10 |  |  |
|  | XP\_065062463\_9914-model12 | -17.9081 |
|  | XP\_065062463\_9914-model12.1 | -17.6678 |
|  | XP\_065062463\_9914-model12.2 | -17.1345 |
|  | XP\_065062463\_9914-model12.3 | -16.9705 |
|  | XP\_065062463\_9914-model12.4 | -16.9539 |
|  | XP\_065062463\_9914-model12.5 | -16.7762 |
|  | XP\_065062463\_9914-model12.6 | -16.7687 |
|  | XP\_065062463\_9914-model12.7 | -16.686 |
|  | XP\_065062463\_9914-model12.8 | -16.4036 |
|  | XP\_065062463\_9914-model12.9 | -16.2639 |
| # cluster 13 - Size: 1 |  |  |
|  | XP\_065062463\_9914-model13 | -17.875 |
| # cluster 14 - Size: 6 |  |  |
|  | XP\_065062463\_9914-model14 | -17.5815 |
|  | XP\_065062463\_9914-model14.1 | -17.5157 |
|  | XP\_065062463\_9914-model14.2 | -17.4302 |
|  | XP\_065062463\_9914-model14.3 | -17.2895 |
|  | XP\_065062463\_9914-model14.4 | -16.9558 |
|  | XP\_065062463\_9914-model14.5 | -16.6653 |
| # cluster 15 - Size: 5 |  |  |
|  | XP\_065062463\_9914-model15 | -17.2678 |
|  | XP\_065062463\_9914-model15.1 | -17.2512 |
|  | XP\_065062463\_9914-model15.2 | -17.1199 |
|  | XP\_065062463\_9914-model15.3 | -16.6567 |
|  | XP\_065062463\_9914-model15.4 | -16.5819 |
| # cluster 16 - Size: 2 |  |  |
|  | XP\_065062463\_9914-model16 | -17.1605 |
|  | XP\_065062463\_9914-model16.1 | -16.7954 |
| # cluster 17 - Size: 1 |  |  |
|  | XP\_065062463\_9914-model17 | -16.7091 |
|  |  |  |
